# The Dual Descriptor Reveals the Janus–Faced Behaviour of Diiodine

**DOI:** 10.3389/fchem.2022.869110

**Published:** 2022-03-24

**Authors:** Jorge I. Martínez-Araya

**Affiliations:** Departamento de Ciencias Químicas, Facultad de Ciencias Exactas, Universidad Andres Bello (UNAB), Santiago, Chile

**Keywords:** dual descriptor, second-order fukui function, Janus-faced ligand, bent “end-on” coordination mode, linear “end-on” coordination mode, diiodine molecule

## Abstract

The Janus–faced ligand behavior of diiodine (I_2_) was evidenced after applying the dual descriptor (DD or second-order Fukui function), thus providing additional support to the work performed by Rogachev and Hoffmann in 2013. Along with its capacity to reveal sites susceptible to undergo attacks simultaneously of nucleophilic and electrophilic types, another advantage of DD lies in being an orbital-free descriptor. That means it is based only upon total electron densities when written in its most accurate operational formula. This quality is not exclusive of DD because when Fukui functions are written in terms of electron densities instead of densities of frontier molecular orbitals, they become orbital-free descriptors too. Furthermore, the present work is an application of the generalized operational formula of the dual descriptor published in 2016 that takes into account any possible degeneracy in frontier molecular orbitals. As a proof about capabilities of DD, the possible sites for a favorable interaction between I_2_ with two organometallic compounds [Rh_2_(O_2_CCF_3_)_4_] and [(C_8_H_11_N_2_)Pt (CH_3_)] were correctly revealed by overlapping the biggest lobe for receiving nucleophilic attacks of one molecule with the biggest lobe for receiving electrophilic attacks of the other molecule, so allowing to predict the same coordination modes as experimentally known: linear “end–on” for the [(C_8_H_11_N_2_)Pt (CH_3_)]…I_2_, and bent “end–on” for the [Rh_2_(O_2_CCF_3_)_4_]…I_2_ interactions.

## 1 Introduction

The so–called dual descriptor [DD or second order Fukui function] symbolized as *f* ^(2) ^(**r**) was proposed by Morell and coworkers (Morell et al. (2005; 2006)). DD as able to reveal those sites in a molecule that can undergo a nucleophilic attack when *f* ^(2) ^(**r**) > 0 or an electrophilic attack when *f* ^(2) ^(**r**) < 0. Because it is a scalar field, it can represented as a 3D picture. Its main advantage lies in the fact that it provides more accurate information about local reactivities than the well–known Fukui functions. This fact is due to an arithmetic cancelation of relaxation terms when the electron density is expressed as a linear combination of molecular orbital densities (Martínez-Araya (2015)). However, degeneracy in frontier molecular orbitals exerts an influence on local reactivity. When ignored, it leads to erroneous local reactivity information. Such a problem was addressed in 2009, but under the frontier molecular orbital approximation (Martínez (2009)). Hence, an obvious question arises: In practice, how can we be accurate enough to yield 3D pictures of the dual descriptor based on its orbital-free operational formula? In other words, how can any possible degeneracy in frontier molecular orbitals be considered without explicitly resorting to them but only dealing with total electron densities? The answer was published in 2016 through an accurate operational formula applied on closed–shell systems (Martínez-Araya (2016)) to obtain 3D pictures of the dual descriptor. Said operational formula is based on total electronic densities (this method is called finite difference approximation, FDA, since the number of electrons is a discrete variable, thus preventing the exact arithmetic definition of a derivative as demanded by DD) while considering any possible degeneracy in frontier molecular orbitals as given by Eq. 1. Since it includes degrees of the degeneracy of HOMO and LUMO given by the integer numbers q and p, respectively, this operational formula is considered an improved version of the original one (Morell et al. (2005)), thus making it more suitable to describe local reactivity:

$$f^{\left(2\right)}\left(r\right)=\frac{q\cdot \rho {\left(r\right)}_{N+p}^{p+1}-\left(p+q\right)\cdot \rho {\left(r\right)}_{N}^{1}+p\cdot \rho {\left(r\right)}_{N-q}^{q+1}}{p\cdot q}.$$ (1)

where $\rho {\left(r\right)}_{N+p}^{p+1}$ is the electron density of the molecular system bearing *N* + p electrons; $\rho {\left(r\right)}_{N}^{1}$ is the electron density of the original molecular system bearing *N* electrons; $\rho {\left(r\right)}_{N-q}^{q+1}$ is the electron density of the molecular system bearing *N* − q electrons. Superscripts (p + 1, 1, and q + 1) indicate the spin-multiplicity associated with each electron density, so that they are not powers. And subscripts (*N* + p, *N*, and *N* − q) correspond to the total number of electrons associated to the respective electron density. Besides, p and q (the degrees of degeneracy of the LUMO and HOMO respectively), also indicate the number of hypothetical arriving electrons to the p–fold LUMO and leaving electrons from the q–fold HOMO.

Rogachev and Hoffmann (Rogachev and Hoffmann (2013)) found that the diiodine molecule can react as an electron–acceptor or as an electron–donor depending on its counterpart. On the one hand, for instance, they discovered that the diiodine behaves as an electron–acceptor through a σ*(I-I) orbital when interacting with the Pt atom of the [(C_8_H_11_N_2_)Pt (CH_3_)]. On the other hand, they also reported that diiodine behaves as an electron–donor towards the Rh_2_–core of [Rh_2_(O_2_CCF_3_)_4_] through the σ*(Rh-Rh) orbital. In the present work, the same conclusion concerning the double behavior of diiodine molecule is reached when using the operational formula given by Eq. 1; which has the advantage of not using molecular orbital theory due to its direct dependence upon total electronic densities $\rho {\left(r\right)}_{N+p}^{p+1}$ , $\rho {\left(r\right)}_{N}^{1}$ , and $\rho {\left(r\right)}_{N-q}^{q+1}$ .

## 2 Methods

For this work, the software Gaussian16 was used (Frisch et al. (2016)). From the Basis Set Exchange website (https://www.basissetexchange.org/), the def2–QZVPPD basis set (Rappoport and Furche (2010)), which includes diffuse functions for all atoms involved in the present work along with pseudo–potentials for the I, Pt, and Rh atoms, was selected and employed. Diffuse functions are mandatory to get a correct description of local reactivity given by the dual descriptor as demonstrated in a previous work (Martínez-Araya (2019)). Gusev suggested the use of the M06–L density functional (Gusev (2013)) because it is accurate enough to compute thermodynamic parameters of chemical reactions involving organometallic and metal–organic complexes based on transition metals as the ones analyzed here. As a consequence, the M06–L density functional was employed in the present work.

It is important to clarify that for the Pt and Rh complexes, coordinates for the optimized geometry were obtained from the data published by Rogachev and Hoffman. Geometry optimizations were done solely on the diiodine molecule in the gas phase. The route section for the optimization job was set as follows: M06L/GenECP SCF=(Tight, Fermi,MaxCyc = 200,NoVarAcc,XQC) Int =(Grid = Ultrafine,acc2e = 12) Guess = Huckel Opt=(VeryTight, CalcAll,MaxCyc = 300). Nevertheless, single point calculations in the gas phase were done on I_2_, [(C_8_H_11_N_2_)Pt (CH_3_)], and [Rh_2_(O_2_CCF_3_)_4_] according to the following route section: M06L/GenECP NoSymm SCF=(VeryTight, Fermi,maxcyc = 200,NoVarAcc,XQC) Int=(Grid = Ultrafine,acc2e = 12). Notice that no symmetry restriction was set for all single-point calculations to freeze the Cartesian coordinates system, which is an essential procedure to build up the 3D pictures of scalar fields through the Gaussian software package. Pure d functions were added to the diiodine molecule through the option 5D.

The commands specified for the SCF either speed–up or guarantee convergence of the SCF cycles. Firstly, tight sets the energy convergence criterion to 10^–8^ Hartree. The option Fermi speeds up the convergence of the SCF cycles by broadening the gap between virtual and occupied orbitals by means of fractionally occupied orbitals around the Fermi energy during the SCF cycles (Rabuck and Scuseria (1999)). The maximum number of SCF cycles was set to 200 to assure the convergence criterion is met and NoVarAcc maintains a high accuracy in the calculations from the very beginning of calculations, as opposed to the default option which is that the accuracy of the calculations increases with each step. Finally, XQC allows for a quadratic convergence of the SCF (Bacskay (1981)); therefore speeding–up the process. The initial parameters (guess) for the SCF calculations were obtained with the Hückel method, which was indicated to Gaussian with Guess = Huckel.

For the integration process, setting the grid parameter to ultrafine (Krack and Köster (1998)) means that, using a minimal number of points, integration grids achieve a level of accuracy of 99 radial shells and 590 points per shell (Lebedev and Skorokhodov (1992)). In addition, imposing acc2e = 12 sets two–electron integral accuracy parameter to 10^–12^ Hartree. On the other hand, the convergence accuracy for the geometrical optimization calculations was set at 0.000002 for maximum force, 0.000001 for RMS (root mean square), 0.000006 for maximum displacement, and 0.000004 for RMS displacement with the VeryTight command. The CalcAll option in the Opt parenthesis computes the force constant at every point and with MaxCyc the maximum number of geometrical optimization steps was set to 300.

In order to work with Eq. 1, electron densities must be obtained from the single point calculations. This was achieved using the cubegen program, which constructed 3D pictures of the dual descriptor to yield the electron densities. The arithmetic operations between these densities were carried out using the cubman program. Both programs are included in the Gaussian 16 software package (Frisch et al. (2016)).

The values for the different parameters of Eq. 1 were set in accordance with the chemical nature of each species. For example, for the diiodine molecules *N* = 106 (the total number of electrons), p = 1 and q = 2. The values of p and q follow Hund’s maximum multiplicity rule and take into consideration, respectively, the non-degenerate LUMO and doubly degenerate HOMO of the diiodine molecule (Table 2). Spin multiplicities are therefore set to 2 (coming from p + 1) and 3 (coming from q + 1). Eq. 1 then turns into Eq. 2:

$$f^{\left(2\right)}\left(r\right)=\frac{2\cdot \rho {\left(r\right)}_{107}^{2}-3\cdot \rho {\left(r\right)}_{106}^{1}+\rho {\left(r\right)}_{104}^{3}}{2}.$$ (2)

Because 28 electrons per iodine atom are replaced with the pseudopotentials included in the def2-Q ZVPPD basis set, the total number of electrons dealt with is 50 (*N* = 106–2 ⋅ 28 = 50), leading to the following definitive operational formula:

$$f^{\left(2\right)}\left(r\right)=\frac{2\cdot \rho {\left(r\right)}_{51}^{2}-3\cdot \rho {\left(r\right)}_{50}^{1}+\rho {\left(r\right)}_{48}^{3}}{2}.$$ (3)

An energetic threshold must be set by which to define degeneracy and hence the values for the variables p and q in Eq. 1 for the Rh and Pt complexes owing to the closeness among energies of molecular orbitals of transition metal complexes (quasi–degeneracy). It seems reasonable to consider a degeneracy case when the energy difference between two neighboring molecular orbitals is equal or smaller than the 2% of the HOMO–LUMO energy gap. That percentage is based on the scientific experience of the author when dealing with cases of quasi-degenerate orbitals; therefore, it could vary depending on the user’s criterium, but not much more than 5% as a suggestion. The author’s pendant task is to conclude an appropriate percentage to address these quasi–degenerate orbitals cases, which implies studying several organometallic and metal–organic systems, which is not the goal of the present work. Under this restriction the values for p and q have been set for both the Pt and the Rh complexes. As a consequence, like done on the diiodine molecule, the same analysis has been performed on [(C_8_H_11_N_2_)Pt (CH_3_)] and [Rh_2_(O_2_CCF_3_)_4_]. Two operational formulae of dual descriptor are inferred from Eq. 1 since 60 electrons are replaced by a pseudopotential for the Pt atom in the [(C_8_H_11_N_2_)Pt (CH_3_)] complex (*N* = 160 − 60 = 100), and 28 electrons are also replaced by a pseudopotential for each Rh atom in the [Rh_2_(O_2_CCF_3_)_4_] complex (*N* = 310 − 2 ⋅ 28 = 254), the following operational formulae are obtained:

For the [(C_8_H_11_N_2_)Pt (CH_3_)] complex

$$f^{\left(2\right)}\left(r\right)=\frac{2\cdot \rho {\left(r\right)}_{102}^{3}-4\cdot \rho {\left(r\right)}_{100}^{1}+2\cdot \rho {\left(r\right)}_{98}^{3}}{4}.$$ (4)

For the [Rh_2_(O_2_CCF_3_)_4_] complex

$$f^{\left(2\right)}\left(r\right)=\rho {\left(r\right)}_{255}^{2}-2\cdot \rho {\left(r\right)}_{254}^{1}+\rho {\left(r\right)}_{253}^{2}.$$ (5)

Although the local reactivity analyses exposed in the present work are based on quantum chemical calculations at the M06–L/def2–QZVPPD level of theory, owing to 53% of electrons of I_2_ is replaced with pseudopotentials (28 inner electrons per iodine atom), an additional all–electron single point quantum chemical calculation only for I_2_ was performed at the M06–L/UGBS1p level of theory with the optimized geometry obtained at the M06-L/def2-QZVPPD level of theory (meaning M06–L/UGBS1p//M06–L/def2–QZVPPD) to discard a possible result that could be an artifact due the use of pseudopotentials for iodine included in the def2–QZVPPD basis set. UGBS1p corresponds to the universal Gaussian basis set developed by de Castro, Jorge, and coworkers, including an additional 1p polarization function for each function in the normal UGBS basis set (Silver et al. (1978); Silver and Nieuwpoort (1978); Mohallem et al. (1986); Mohallem and Trsic (1987); da Costa et al. (1987); da Silva et al. (1989); Jorge et al. (1997b,a); de Castro and Jorge (1998)). This type of calculation requested a Douglas–Kroll–Hess second order scalar relativistic method (Douglas and Kroll (1974); Hess (1985, 1986); Jansen and Hess (1989); Barysz and Sadlej (2001); de Jong et al. (2001); Visscher and Dyall (1997)), thus allowing to employ the Eq. 2 for diiodine.

## 3 Results and Discussion

After geometrical optimization at the M06–L/def2–QZVPPD level of theory, the interatomic distance is comparable to the experimental one (a 0.7% of error with respect to the experimental iodine–iodine distance). Therefore, it can be stated that the level of theory employed reproduces the bond length within an acceptable accuracy: 2.69Å (the experimental value is 2.67Å (Rumble (2019)).

**FIGURE 1:**
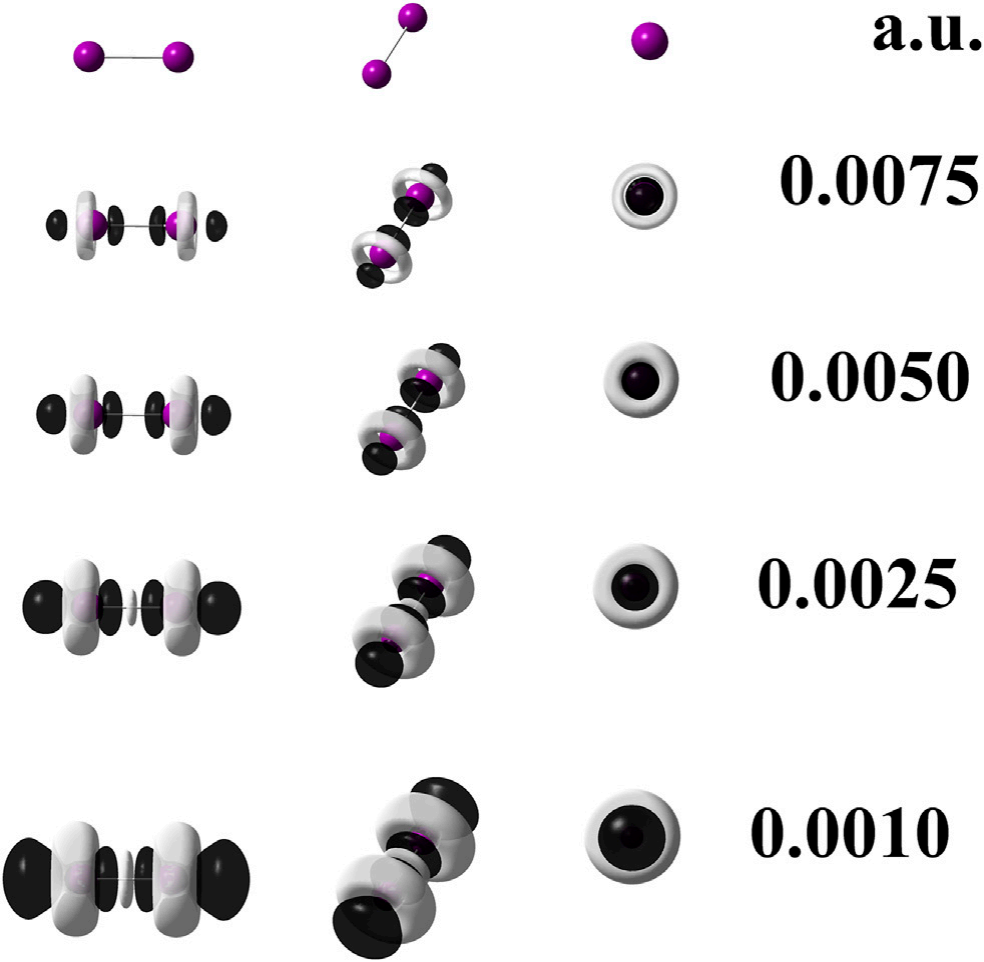
Dual descriptor (DD) of diiodine computed at the M06–L/UGBS1p//M06–L/def2-QZVPPD level of theory at different isovalues given in atomic units. To use Eq. 2, the following total electron densities were employed: $\rho {\left(r\right)}_{107}^{2}$ , $\rho {\left(r\right)}_{106}^{1}$ , and $\rho {\left(r\right)}_{104}^{3}$ . Purple spheres stand for iodine atoms, black–colored lobes represent positive values of DD (*f* ^(2) ^(**r**)>0); white–colored lobes correspond to negative values of DD (*f* ^(2) ^(**r**)<0).

The calculation at the M06–L/UGBS1p//M06–L/def2–QZVPPD level of theory carried out only for diiodine provides information to use the Eq. 2. It was applied to check whether the explicit presence of all electrons could influence or not differently on the local reactivity given by the dual descriptor when compared against the result provided by the single point calculation at the M06–L/def2–QZVPPD level of theory. The degeneracy manifested by the HOMO and HOMO-1 orbitals in the diiodine molecule, as quoted in Table 1, leads to q = 2. On the other hand, there is no degeneracy regarding the LUMO, and as a consequence, p = 1. Figure 1 depicts the dual descriptor for diiodine obtained at the M06–L/UGBS1p//M06–L/def2–QZVPPD level of theory. This 3D picture can be considered a reference figure for the dual descriptor of diiodine.

**TABLE 1:** Energies (in Hartree) of molecular orbitals in the diiodine molecule at the M06–L/UGBS1p//M06–L/def2–QZVPPD level of theory.

Orbital	Energy
LUMO +2	0.083 18
LUMO +1	0.048 97
LUMO	−0.163 04
HOMO	−0.230 23
HOMO -1	−0.230 23
HOMO -2	−0.279 90

Furthermore, in order to check whether frontier molecular orbitals are the main responsible for the local reactivity of diiodine or not, the dual descriptor was also generated through its operational formula based on the frontier molecular orbital approximation (FMOA) as given in the respective reference (Martínez (2009); Martínez et al. (2010); Martínez-Araya (2012b,a)). Such as operational formula based on the densities of frontier molecular orbitals is written as follows:

$$f^{\left(2\right)}\left(r\right)={\left|\Psi_{\text{LUMO}}\left(r\right)\right|}^{2}-\frac{1}{2}\left\{{\left|\Psi_{\text{HOMO}}\left(r\right)\right|}^{2}+{\left|\Psi_{\text{HOMO}\;-1}\left(r\right)\right|}^{2}\right\}.$$ (6)

**FIGURE 2:**
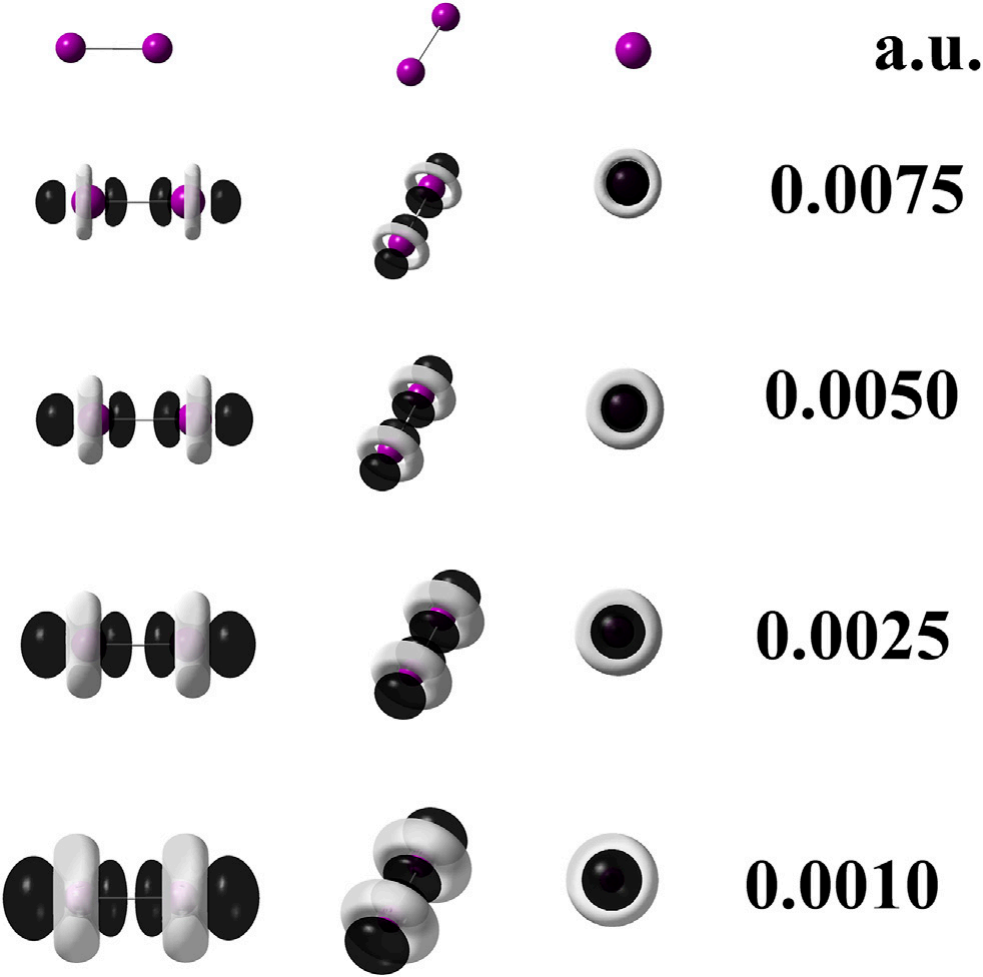
Dual descriptor (DD) of diiodine computed at the M06–L/UGBS1p//M06–L/def2-QZVPPD at different isovalues given in atomic units. To use Eq. 6, the following densities of frontier molecular orbitals were employed: ${\left|\Psi_{\text{LUMO}}\left(r\right)\right|}^{2}$ , ${\left|\Psi_{\text{HOMO}}\left(r\right)\right|}^{2}$ , and ${\left|\Psi_{\text{HOMO}\;-1}\left(r\right)\right|}^{2}$ . Purple spheres stand for iodine atoms, black–colored lobes represent positive values of DD (*f* ^(2) (^ **r**)>0); white–colored lobes correspond to negative values of DD (*f* ^(2) (^ **r**)<0).

Figure 2 displays the dual descriptor according to Eq. 6. As can be observed, there is no substantial difference when compared against Figure 1. Comparing both scalar fields, the one given by Eq. 2 and that given by Eq. 6 allows us to infer that frontier molecular orbitals rule the local reactivity of I_2_.

**FIGURE 3:**
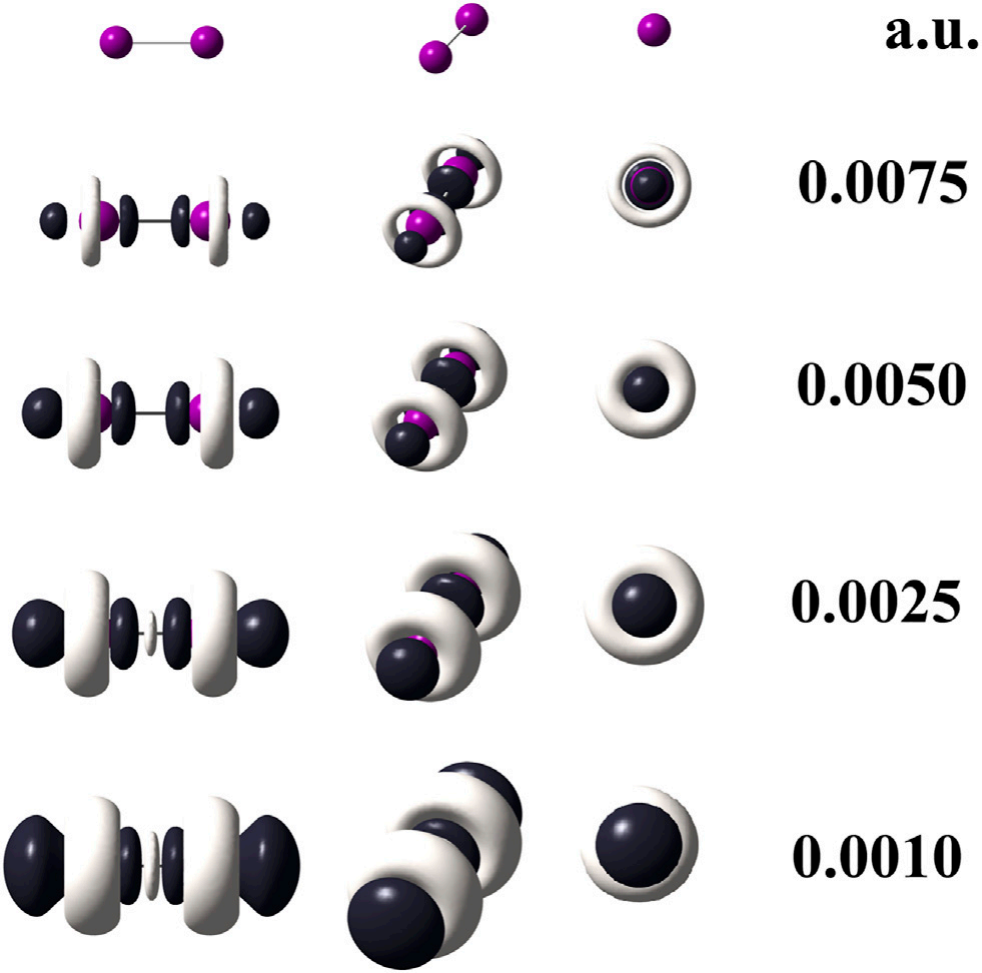
Dual descriptor (DD) of diiodine computed at the M06–L/def2-QZVPPD at different isovalues given in atomic units. To use Eq. 3, the following total electron densities were employed: $\rho {\left(r\right)}_{51}^{2}$ , $\rho {\left(r\right)}_{50}^{1}$ , and $\rho {\left(r\right)}_{48}^{3}$ . Purple spheres stand for iodine atoms, black–colored lobes represent positive values of DD (*f* ^(2) ^(**r**)>0); white–colored lobes correspond to negative values of DD (*f* ^(2) ^(**r**)<0).

**FIGURE 4:**
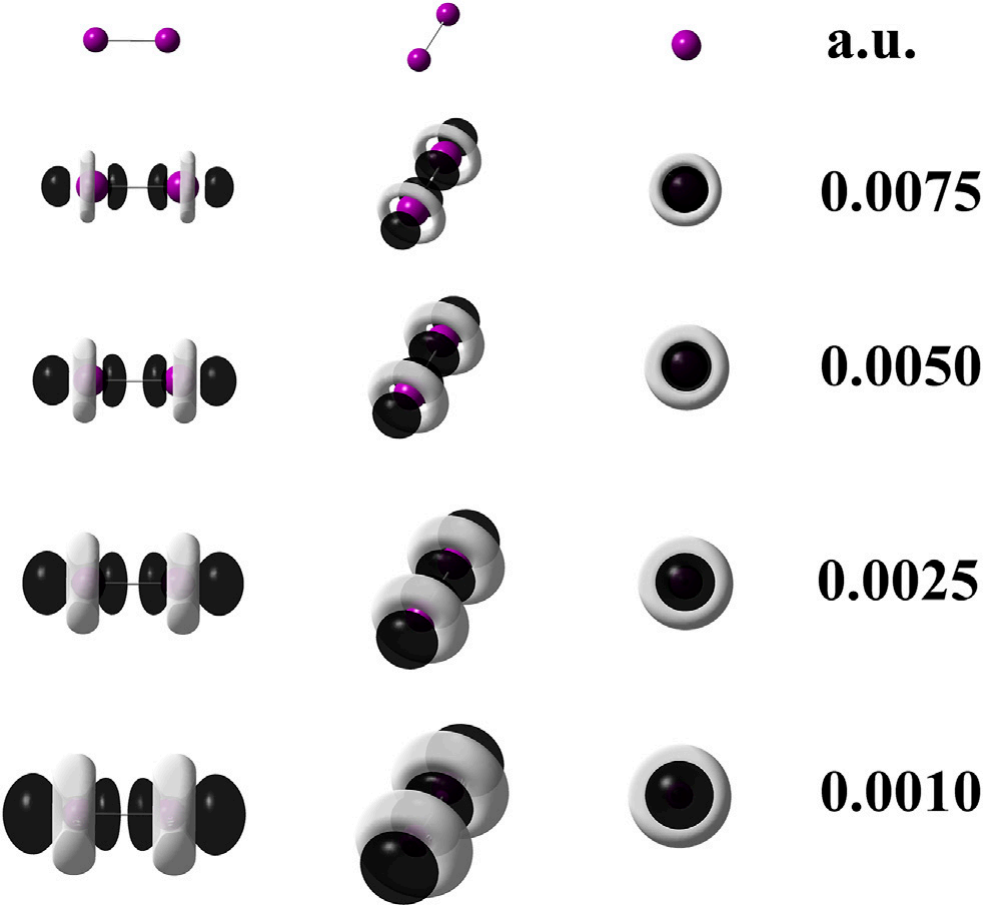
Dual descriptor (DD) of diiodine computed at the M06–L/def2-QZVPPD at different isovalues given in atomic units. To use Eq. 6, the following densities of frontier molecular orbitals were employed: ${\left|\Psi_{\text{LUMO}}\left(r\right)\right|}^{2}$ , ${\left|\Psi_{\text{HOMO}}\left(r\right)\right|}^{2}$ , and ${\left|\Psi_{\text{HOMO}\;-1}\left(r\right)\right|}^{2}$ . Purple spheres stand for iodine atoms, black–colored lobes represent positive values of DD (*f* ^(2) ^(**r**)>0); white–colored lobes correspond to negative values of DD (*f* ^(2) ^(**r**)<0).

When quantum chemical calculations at the M06–L/def2–QZVPPD level of theory are performed for I_2_, the use of Eq. 3 is mandatory because it implies that pseudopotentials replaced 56 electrons in the I_2_ molecule. Hence, to confirm this statement, the Eq. 6 was applied to generate the dual descriptor as depicted by Figure 4 which shows an expected resemblance with Figure 3. The degeneracy exhibited by the HOMO and HOMO-1 orbitals for the diiodine molecule implies q = 2 as inferred from Table 2. Figure 3 indicates that orbital relaxation is negligible because inner orbitals do not exert an influence on local reactivity. So that for diiodine, the FMOA should be accurate enough to assess its possible local interactions with other molecules through the use of the operational formula given by Eq. 6. Hence, to confirm this statement, the Eq. 6 was applied so generating the dual descriptor as depicted by Figure 4 which shows an evident resemblance with Figure 3. As a consequence, the use of the operational formula given by Eq. 3 for diiodine is accurate enough to analyze its possible covalent interactions with the [(C_8_H_11_N_2_)Pt (CH_3_)] and [Rh_2_(O_2_CCF_3_)_4_] organometallic compounds through their operational formulae given by Eqs 4, 5, respectively.

**TABLE 2:** Energies (in Hartree) of molecular orbitals in the diiodine molecule at the M06–L/def2–QZVPPD level of theory.

Orbital	Energy
LUMO +2	0.010 97
LUMO +1	0.007 90
LUMO	−0.146 33
HOMO	−0.225 70
HOMO -1	−0.225 70
HOMO -2	−0.285 21

**FIGURE 5:**
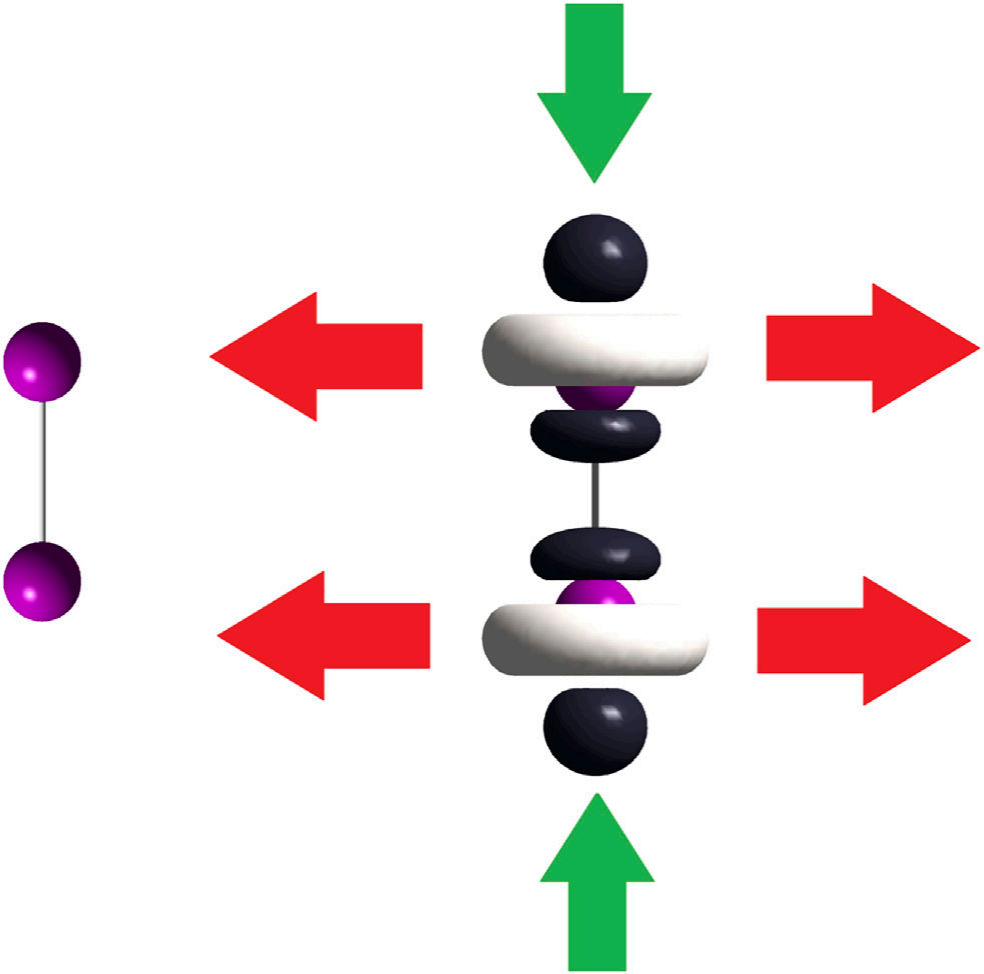
On the left side, the iodine molecule; on the right side, dual descriptor of this molecule. Green arrows indicate the electron–acceptor (electrophilic) regions leading to a linear “end–on” coordination mode and red arrows show us the electron–donor (nucleophilic) regions thus leading to a bent “end–on” coordination mode.

Following the 2% threshold criterium, a two-fold HOMO and two-fold LUMO are presented in the [(C_8_H_11_N_2_)Pt (CH_3_)] compound. The doubly degenerate frontier orbitals imply p = q = 2. On the contrary, there is no degeneracy of the frontier molecular orbitals of [Rh_2_(O_2_CCF_3_)_4_], in which case p and q equal 1. Energies of the nearest two virtual and two occupied molecular orbitals to the LUMO and HOMO, are quoted in Table 3 for [(C_8_H_11_N_2_)Pt (CH_3_)] and Table 4 for [Rh_2_(O_2_CCF_3_)_4_]. Different cuts of the dual descriptor are displayed for the diiodine molecule in Figure 3. According to the color code used here, Figure 5 indicates the electron flow coming in (green arrows) and going out (red arrows) from this molecule. Dark–colored lobes located at the upper and lower axial positions represent the electrophilic regions, while white–colored lobes surrounding iodine atoms are the nucleophilic regions. Notice that red arrows should be drawn all around both iodine atoms on planes perpendicular to the chemical bond, as if forming a disk around each iodine atom. However, for the sake of clarity only two red arrows per each imaginary plane have been drawn.

**TABLE 3:** Energies (in Hartree) of molecular orbitals in [(C_8_H_11_N_2_)Pt (CH_3_)] at the M06–L/def2–QZVPPD level of theory.

Orbital	Energy
LUMO +2	−0.007 23
LUMO +1	−0.020 63
LUMO	−0.022 37
HOMO	−0.158 81
HOMO -1	−0.160 52
HOMO -2	−0.177 34

**TABLE 4:** Energies (in Hartree) of molecular orbitals in the [Rh_2_(O_2_CCF_3_)_4_] at the M06–L/def2–QZVPPD level of theory.

Orbital	Energy
LUMO +2	−0.124 89
LUMO +1	−0.140 26
LUMO	−0.183 31
HOMO	−0.226 17
HOMO -1	−0.228 53
HOMO -2	−0.228 63

**FIGURE 6:**
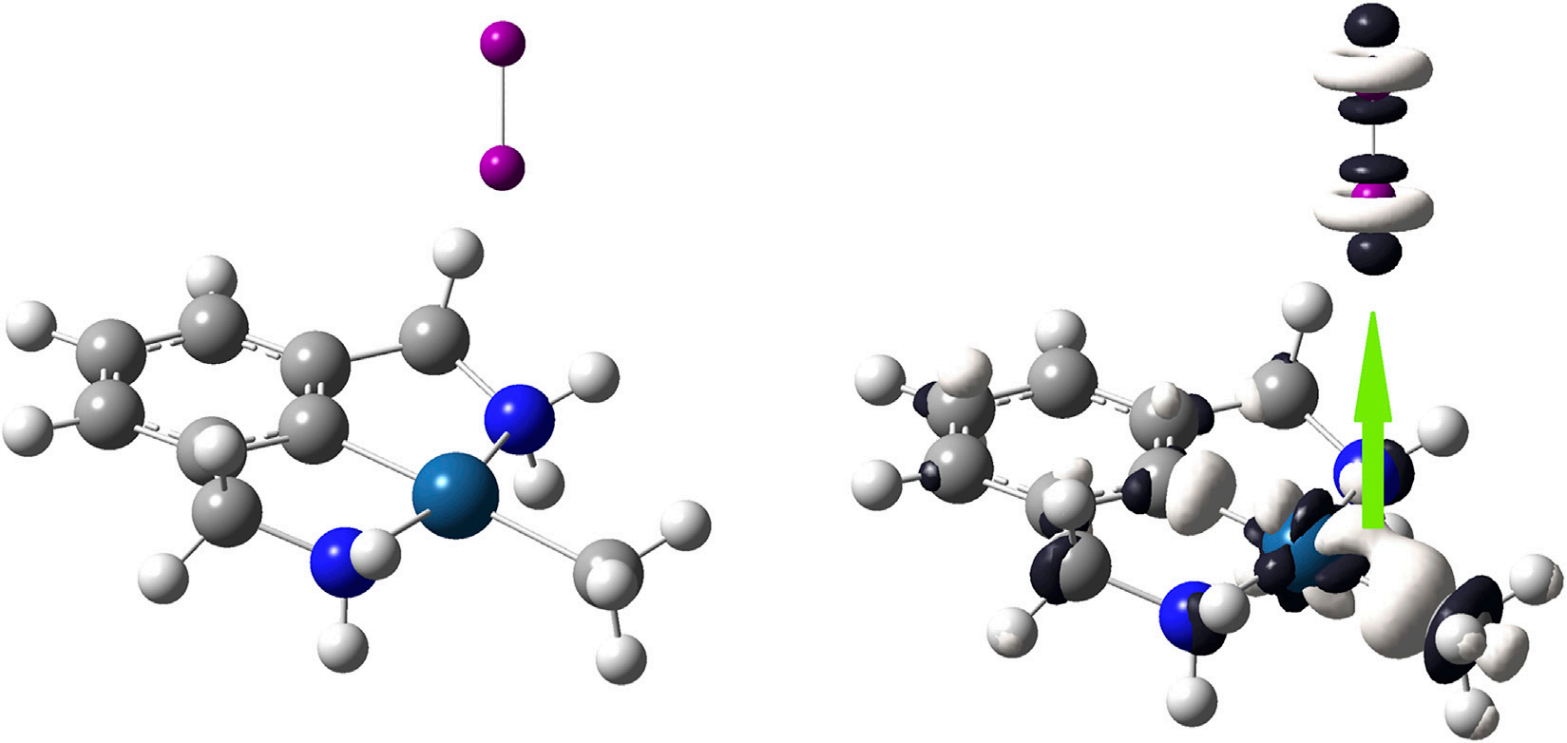
On the left side, the [(C_8_H_11_N_2_)Pt (CH_3_)] molecule; on the right side, dual descriptor of this transition metal complex is displayed at an isovalue of 0.010 a. u. in order to highlight that the nearest region to the Pt atom is the most reactive to donate electrons; dual descriptor of diiodine is displayed at an isovalue of 0.0050 a. u. Overlapping one of the biggest lobes for receiving nucleophilic attacks onto I_2_ (one of the dark–colored lobes) with the biggest lobe for receiving an electrophilic attack onto [(C_8_H_11_N_2_)Pt (CH_3_)] (the white–colored lobe). This favorable covalent interaction between these opposite lobes leads to a linear “end-on” coordination mode.

Figure 6 shows that the favorable interaction occurs through the Pt atom in such a way that this atom donates electrons towards one of the two atoms of diiodine. As observed, the most significative white–colored lobe of the Pt–based complex is located on the Pt atom, making it the most susceptible to undergo an electrophilic attack from the diiodine molecule through its electrophilic axial region represented by dark–colored lobes. Clearly the Pt–based complex reacts as a nucleophilic species and the diiodine molecule approaches perpendicularly on the plane of this Pt–based complex, so leading to the linear “end–on” coordination mode.

**FIGURE 7:**
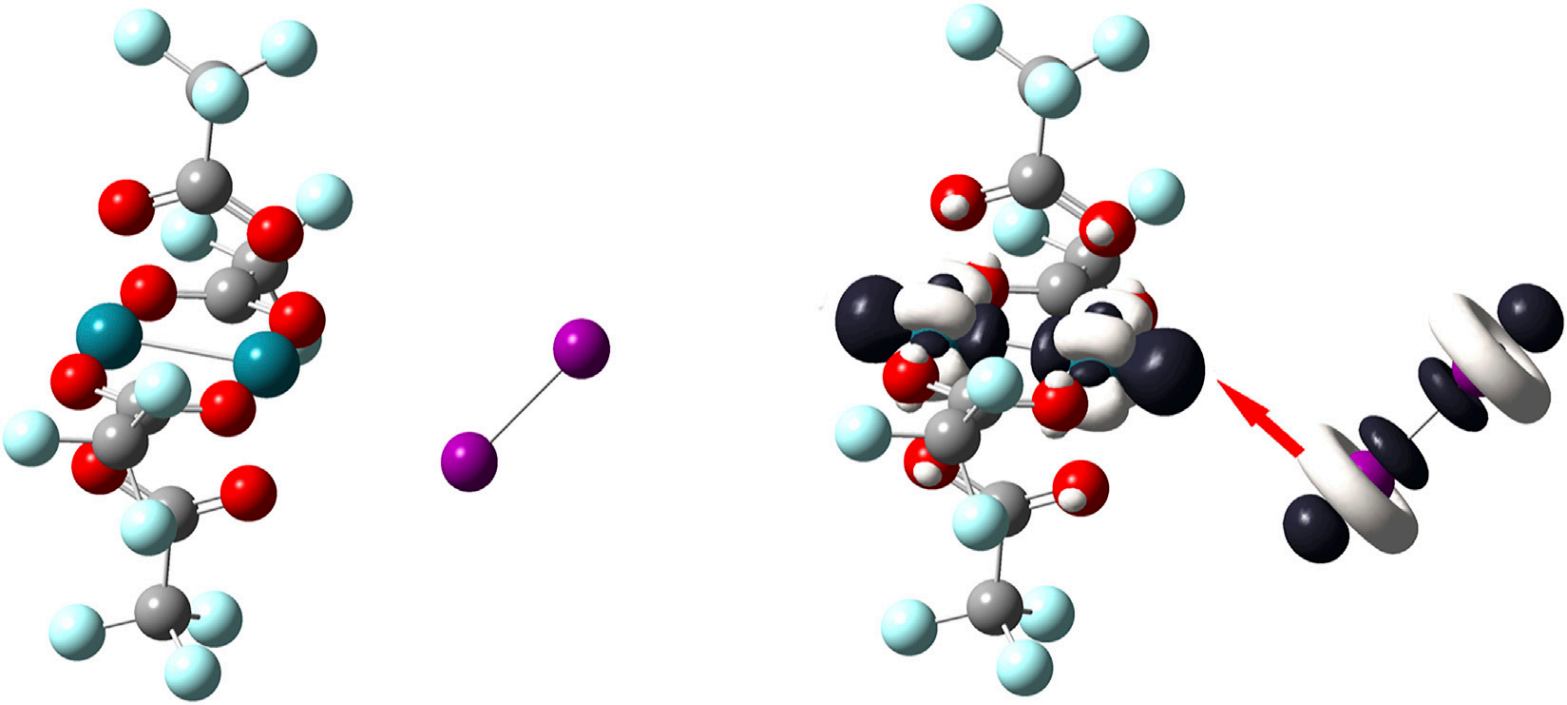
On the left side, the [Rh_2_(O_2_CCF_3_)_4_] molecule; on the right side, dual descriptor of this transition metal complex is displayed at an isovalue of 0.010 a. u. so that Rh atoms are the most reactive to accept electrons; dual descriptor of diiodine is displayed at an isovalue of 0.0050 a. u. Overlapping one of the biggest lobes for receiving electrophilic attacks onto I_2_ (one of the white–colored lobes) with one of the biggest lobes for receiving a nucleophilic attack onto [Rh_2_(O_2_CCF_3_)_4_] (one of the dark–colored lobes). This favorable covalent interaction between these opposite lobes leads to a bent “end–on” coordination mode.

The opposite situation is depicted by Figure 7. In this case, the most reactive regions on the Rh–based complex are located around both metal atoms. According to the dual descriptor, these atoms are susceptible to undergo nucleophilic attacks and since the diiodine molecule is again the countpart molecule to react it uses its nucleophilic regions; represented by the white–colored lobes that equatorially surround the iodine atoms. Thanks to one of these nucleophilic lobes, the diiodine molecule approaches side by side towards the Rh–based complex so leading to the bent “end–on” coordination mode. Notice here that the diiodine molecule acts as a molecule that donates electrons towards the closest Rh atom of the metal complex which reacts as an electrophilic species.

## 4 Conclusion

The operational formula of the dual descriptor (DD) adapted to molecular symmetry corresponds to an orbital–free local reactivity descriptor because there is no explicit dependence upon densities of fronter molecular orbitals, thus accomplishing with the essential philosophy of the Conceptual Density–Functional Theory: to explain all local reactivity in terms of electron density and not in terms of molecular orbitals. Furthermore, as an example, DD revealed that the diiodine (I_2_) molecule exhibits an electrophilic behavior when facing [(C_8_H_11_N_2_)Pt (CH_3_)] leading to form a chemical bond of the type linear “end–on” coordination mode. But when I_2_ shows a nucleophilic behavior when facing [Rh_2_(O_2_CCF_3_)_4_] which leads to form a chemical bond of the type bent “end–on” coordination mode. This study agrees with Hoffmann’s theoretical evidence supported by MOT (molecular orbital theory), NBO (natural bond orbital), and EDA (energy decomposition analysis).

### 4.1 Permission to Reuse and Copyright

Figures, tables, and images will be published under a Creative Commons CC-BY licence and permission must be obtained for use of copyrighted material from other sources (including re-published/adapted/modified/partial figures and images from the internet). It is the responsibility of the authors to acquire the licenses, to follow any citation instructions requested by third-party rights holders, and cover any supplementary charges.

## Data Availability

The raw data supporting the conclusion of this article will be made available by the authors, without undue reservation.
